# Transcriptome Analysis of the Inhibitory Effects of 20(S)-Protopanaxadiol on NCI-H1299 Non-Small Cell Lung Cancer Cells

**DOI:** 10.3390/molecules28155746

**Published:** 2023-07-29

**Authors:** Zhongyi Cong, Xinmin Zhang, Zeqi Lv, Jingyuan Jiang, Lei Wang, Jiapeng Li, Jie Wang, Jianjun Zhao

**Affiliations:** 1 Department of Regenerative Medicine, School of Pharmaceutical Science, Jilin University, Fujin Road 1266, Changchun 130021, China; congzhy@jlu.edu.cn (Z.C.); zhangxinmin@jlu.edu.cn (X.Z.); lvzq21@mails.jlu.edu.cn (Z.L.); jiangjy21@mails.jlu.edu.cn (J.J.); wlei22@mails.jlu.edu.cn (L.W.); 17808051780@163.com (J.L.); jiewang2820@mails.jlu.edu.cn (J.W.); 2 Department of Respiratory Medicine, China-Japan Union Hospital of Jilin University, Xiantai Street 126, Changchun 130033, China

**Keywords:** non-small cell lung cancer, protopanaxadiol, transcriptome, transcriptional profile, ERK, p38

## Abstract

Lung cancer seriously threatens human health. To explore the molecular mechanism of 20(S)-Protopanaxadiol (PPD) on human non-small cell lung cancer cells, we investigated the transcriptional profile of PPD-treated NCI-H1299 cells. Cell proliferation, cell cycle, and apoptosis were detected using cell counting kit-8 and flow cytometry, respectively. Differentially expressed genes (DEGs) between PPD-treated and untreated cells were determined using RNA sequencing and bioinformatic analysis. Protein phosphorylation was detected using Western blotting. Data of mRNA expression profiles of lung cancer were from The Cancer Genome Atlas (TCGA) and analyzed using R software version 4.3.1. PPD showed an inhibitory effect on the proliferation of NCI-H1299 cells and induced apoptosis. There were 938 upregulated genes and 466 downregulated genes in PPD-treated cells, and DEGs were primarily enriched in the MAPK signaling pathway. The detection of phosphorylation revealed that the phosphorylation of ERK and p38 MAPK was significantly reduced in PPD-treated cells. Further comparison of PPD-regulated DEGs with clinical data of lung adenocarcinoma demonstrated that most downregulated genes in tumor tissues were upregulated in PPD-treated cells or vice versa. Two PPD-downregulated genes *HSPA2* and *EFNA2* were associated with patients’ overall survival. Therefore, PPD could inhibit NCI-H1299 cells by affecting gene expression and regulating ERK and p38 MAPK pathways.

## 1. Introduction

Lung cancer is the leading cause of cancer death worldwide, with an estimated 1.8 million deaths (18.0%) in 2020. It is the most frequently occurring cancer and the main cause of cancer death in men and ranks third for incidence and second for mortality in women [1]. In China, lung cancer is the most common cancer and the leading cause of cancer death [2]. Several types of lung cancer exist and may roughly be grouped into small-cell lung cancer and non-small cell lung cancer (NSCLC) [3]. NSCLC makes up approximately 85% of all lung cancer cases, which is broken down further into adenocarcinoma, squamous cell carcinoma, and large-cell carcinoma. Among them, adenocarcinoma and squamous cell carcinoma are the two predominant NSCLC histological phenotypes [4]. The main treatment for early-stage NSCLC is surgical resection. Chemotherapy has been used perioperatively to improve the oncologic outcomes of surgery [5]. Nevertheless, early-stage NSCLC is not easily detected. Most patients are at an advanced stage at the time of clinical diagnosis and have lost the opportunity for surgical resection [6]. For decades, the standard of treatment for advanced-stage NSCLC included only palliative cytotoxic chemotherapy with strong toxic side effects leading to poor tolerance in patients and drug resistance of tumor cells for long-term administration [7]. In recent years, targeted therapy and immunotherapy with low side effects have shown excellent outcomes in the treatment of locoregionally advanced and metastatic NSCLC, which specifically inhibit tumor cells [8,9,10]. However, due to the selection of patient indications and high cost, the wide application of these biological therapies is limited [11,12]. The 5-year survival rate of patients with lung cancer is only 10% to 20% in most countries [13]. Therefore, it is necessary to find other anti-lung cancer drugs or methods with high efficiency and low toxicity to expand the treatment options for lung cancer.

The ginseng plant (*Panax ginseng* C.A. Meyer) has been used as a herbal medicine and health food for thousands of years in China and other East Asian countries with multiple biological functions, such as anti-aging, improving cardiovascular diseases, enhancing immunity, and so on [14]. The major pharmacologically active ingredients of ginseng are ginsenosides, which are triterpene saponins. To date, nearly 200 ginsenosides have been identified [15]. Structurally, most of them are composed of a dammarane skeleton of 17 carbons with various sugar moieties attached to the C-3 and C-20 positions, such as Rb1, Rc, Rd, Re, Rf, Rg3, Rh2, etc. These compounds have multifaceted pharmacological activities because of their steroidal structure [16]. In the last few decades, the anti-oxidative, anti-inflammatory, anti-microbial, anti-cardiovascular disease, anti-diabetes, anti-neurological disorder, and anticancer effects have been studied in both basic and clinical research [15,17,18,19]. Biotransformation may be required before ginsenosides become active in mammalian systems. It has been reported that ginsenoside metabolites had greater biological effects than naturally occurring ginsenosides [20,21,22]. 20(S)-Protopanaxadiol (PPD) is one of the major metabolites of ginsenoside following biodegradation with marked inhibitory effects on cells of gastric cancer, colorectal cancer, endometrial cancer, prostate cancer, and lung cancer [16,23,24,25,26,27]. The mechanism of the anti-cancer effect of PPD has not been fully clarified since previous studies generally focused on certain biological processes or signal pathways. In the present study, we demonstrated the inhibitory effect of PPD on human NSCLC NCI-H1299 cells, comprehensively analyzed its effect on tumor cell gene expression at the transcriptional level through transcriptome sequencing, and further explored its mechanism.

## 2. Results

### 2.1. Inhibition Effects of PPD on NCI-H1299 Cells

**Figure 1:**
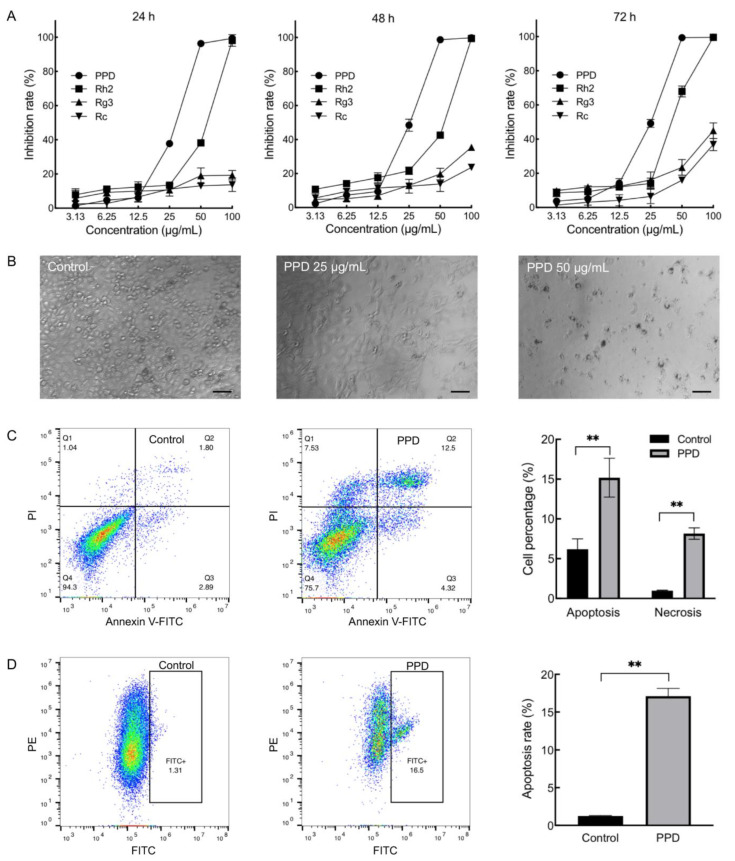
Effects of 20(S)-Protopanaxadiol (PPD) on proliferation and apoptosis of NCI-H1299 cells. (**A**): Cell viability was detected through cell counting kit-8 (CCK-8) assay after treatment by different concentrations (3.13, 6.25, 12.5, 25, 50, and 100 μg/mL) of ginsenosides and PPD for 24, 48, and 72 h. (**B**): Microscope photographs of NCI-H1299 cells treated with 25 and 50 μg/mL of PPD for 72 h (scale bar = 100 μm). (**C**): Cell apoptosis was investigated through flow cytometry (Annexin V-FITC/PI) after PPD (26 μg/mL) treatment for 48 h. **: *p* < 0.01. (**D**): Cell mitochondrial membrane potential was detected through flow cytometry (JC-1) after PPD (26 μg/mL) treatment for 48 h. **: *p* < 0.01.

We compared the effects of PPD on NCI-H1299 cells with extensively studied protopanaxadiol-type ginsenosides Rh2, Rg3, and Rc. In chemical structure, they are all composed of 17 carbon dammarane skeletons but with different sugar moieties attached to the C-3 and C-20 positions (Figure S1). The cells were treated with different concentrations (3.13, 6.25, 12.5, 25, 50, and 100 μg/mL) of ginsenoside and PPD for 24, 48, and 72 h, respectively, the viability of cells was evaluated with cell counting kit-8 (CCK-8), and the inhibition rate was calculated based on the absorbance value of the cells using equation (1). The result showed that the inhibition rate of PPD- (25, 50, and 100 μg/mL) and Rh2- (50 and 100 μg/mL) treated cells were significantly higher than that of the Rg3 and Rc groups (*p* < 0.01, Figure 1A). In addition, the effects of PPD at the concentrations of 25 and 50 μg/mL were stronger than that of Rh2 (*p* < 0.01). Observed under a microscope, the adherent cells decreased in the 25 μg/mL of the PPD-treated group with dead cells suspending in the supernatants, and almost all cells died in 50 μg/mL of the PPD-treated group (Figure 1B). Then we calculated the half maximal inhibitory concentration (IC_50_) of PPD and Rh2 by setting more intensive concentrations (Figure S2). The IC_50_ of PPD on NCI-H1299 cells for 24, 48, and 72 h were 30.86 μg/mL (66.98 μM), 25.97 μg/mL (56.37 μM), and 25.61 μg/mL (55.58 μM), respectively. Moreover, the IC_50_ of Rh2 on NCI-H1299 cells for 24, 48, and 72 h were 54.40 μg/mL (87.34 μM), 50.60 μg/mL (81.24 μM), and 40.34 μg/mL (64.76 μM), respectively. The IC50 of Rg3 and Rc exceeded 100 μg/mL. Therefore, the inhibitory effect of PPD on NCI-H1299 cells was superior to that of Rh2, Rg3, and Rc. We further investigated the effects of PPD (26 μg/mL, 48 h) on cell apoptosis and cell cycle through flow cytometry. The percentage of apoptosis cells (15.18% ± 2.44%) and necrosis cells (8.16% ± 0.71%) of the PPD-treated group were significantly higher than that of the control group (*p* < 0.01, Figure 1C). Cell mitochondrial membrane potential analysis revealed that the percentage of cells with green fluorescence in the PPD-treated group was 17.10% ± 1.04%, which was significantly higher than that of the control group (*p* < 0.01, Figure 1D). However, PPD-treated cells did not show obvious cell cycle arrest compared with the control (Figure S3). The 50% cytotoxic concentration (CC_50_) of PPD acting on NCI-H1299 cells for 48 h was 33.62 μg/mL (72.97 μM) based on cell counting. The results indicated that PPD inhibited the proliferation of NCI-H1299 cells and induced cell apoptosis. In addition, the inhibition rate of PPD (26 μg/mL, 48 h) on human normal fibroblast BJ cells was 16.60%, which was lower than that on NCI-H1299 cells.

### 2.2. Transcriptome Sequencing Analysis of PPD Treated NCI-H1299 Cells

**Figure 2:**
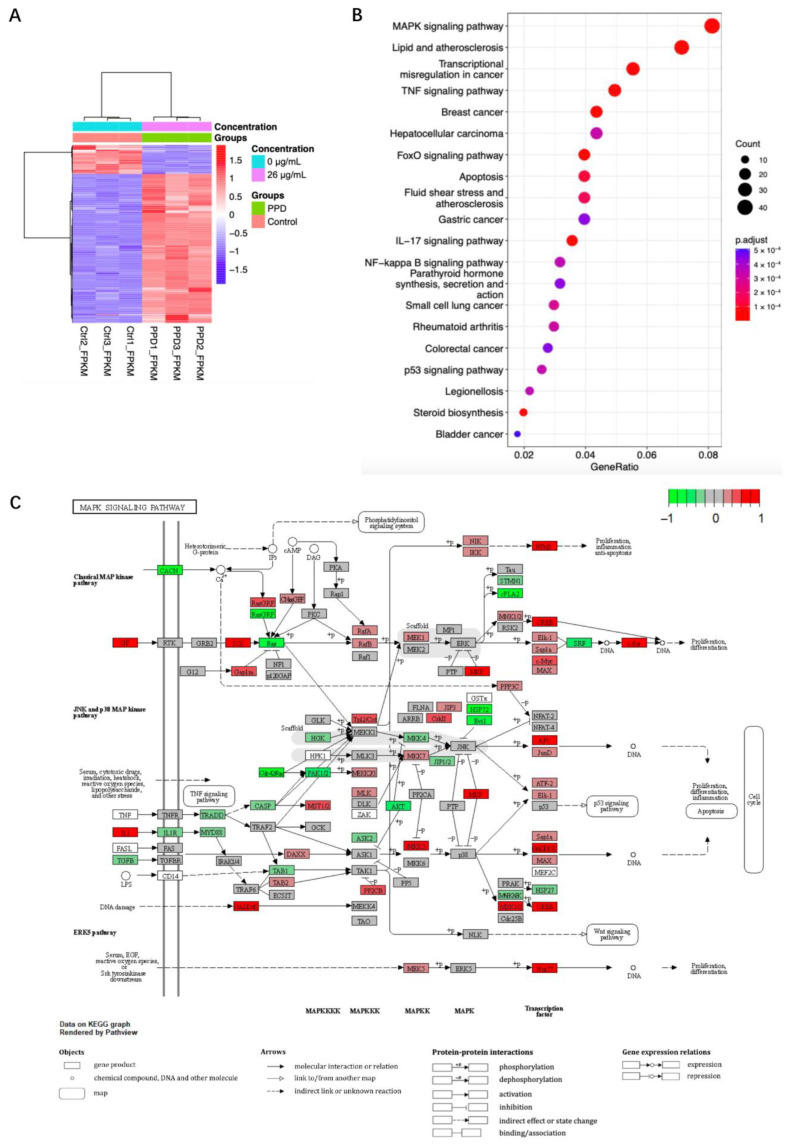
Transcriptome sequencing analysis of PPD-treated NCI-H1299 cells. NCI-H1299 cells were treated with 26 μg/mL of PPD for 48 h followed by transcriptome sequencing analysis. (**A**): Heatmap of differentially expressed genes (DEGs). Colored bars indicate scaled fragments per kilobase million (FPKM) of transcript of each gene (red: High expression, blue: Low expression). Dendrograms represent similarities in clusters of samples (**left**) and in levels of gene expression (**top**). (**B**): Dot plot of enriched Kyoto Encyclopedia of Genes and Genomes (KEGG) pathways. Horizontal axis represents the ratio of DEGs annotated on certain pathway. The vertical axis represents the top 20 pathways with significant enrichment. (**C**): Integration and visualization of gene expression changes on the mitogen-activated protein kinases (MAPK) signaling pathway map. Red represents upregulated genes after normalization, and green represents downregulated genes after normalization.

After being treated with 26 μg/mL of PPD for 48 h, the total RNAs of NCI-H1299 cells were collected for transcriptome sequencing analysis. The value of RNA integrity (RIN) of each sample was approximately 10 (Figure S4) indicating that the quality of RNA was qualified. The raw data of RNA sequencing were submitted to the Sequence Read Archive of the National Center for Biotechnology Information (BioProject ID PRJNA977110). The sequencing depth is shown in Figure S5. R software was used to analyze the data. Compared with the control group, the transcription of 938 genes was upregulated, and 466 genes were downregulated (false discovery rate < 0.05 and |gene expression fold change| > 1.5, Tables S1–S3). Cluster analysis of the differentially expressed genes (DEGs) indicated that genes from the PPD-treated group and the control group were classified into different clusters. The gene expression patterns of the samples in the same group were highly similar, and the gene expression pattern of the PPD group was different from that of the control group (Figure 2A). The DEGs were further analyzed through Kyoto Encyclopedia of Genes and Genomes (KEGG) pathway enrichment analysis to investigate which pathways they were enriched in. Figure 2B showed the top 20 pathways the DEGs were enriched in. Among them, the mitogen-activated protein kinases (MAPK) signaling pathway was ranked first revealing that this pathway contains the most enriched DEGs (Tables S4 and S5). Through R package Pathview, gene expression changes between PPD-treated and untreated samples were further integrated and visualized on the MAPK signaling pathway map (Figure 2C, Table S6). The result indicated that most of the gene expression changes were enriched in the classical extracellular signal-regulated kinase (ERK) MAPK pathway, c-Jun NH_2_-terminal kinase (JNK) MAPK pathway, and p38 MAPK pathway.

### 2.3. Regulation of MAPK in PPD-Treated NCI-H1299 Cells

**Figure 3:**
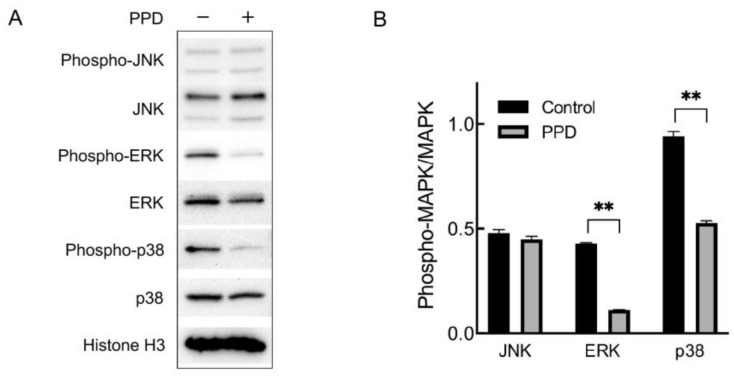
Expression and phosphorylation of MAPKs in PPD-treated NCI-H1299 cells. (**A**): Detection of MAPK expression and phosphorylation through Western blotting. NCI-H1299 cells were treated with 26 μg/mL of PPD for 48 h. (**B**): Gray analysis of Western blotting. **: *p* < 0.01.

Based on the result of the KEGG pathway enrichment analysis that the MAPK signaling pathway enriched in the most DEGs than other pathways, we further investigated whether PPD treatment would regulate the MAPK signaling pathway of NCI-H1299 cell. The mammalian MAPK family consists of ERK, JNK, and p38, which are key kinases phosphorylating various substrate proteins in response to extracellular stimuli. Therefore, we investigated the expression and phosphorylation (activation) of ERK, JNK, and p38. The NCI-H1299 cells were treated with 26 μg/mL of PPD for 48 h followed by a Western blotting assay. As shown in Figure 3, the expression of ERK, JNK, and p38 showed no significant difference between the PPD group and the control group, and the phosphorylation of JNK did not change either. However, the phosphorylation of ERK and p38 was significantly decreased in PPD-treated cells compared with the control (*p* < 0.01), suggesting that PPD might inhibit proliferation and induce apoptosis in NCI-H1299 cells by regulating the ERK and p38 MAPK signaling pathway.

### 2.4. Comparison of PPD-Regulated DEGs with Clinical Data of Lung Adenocarcinoma Patients

**Figure 4:**
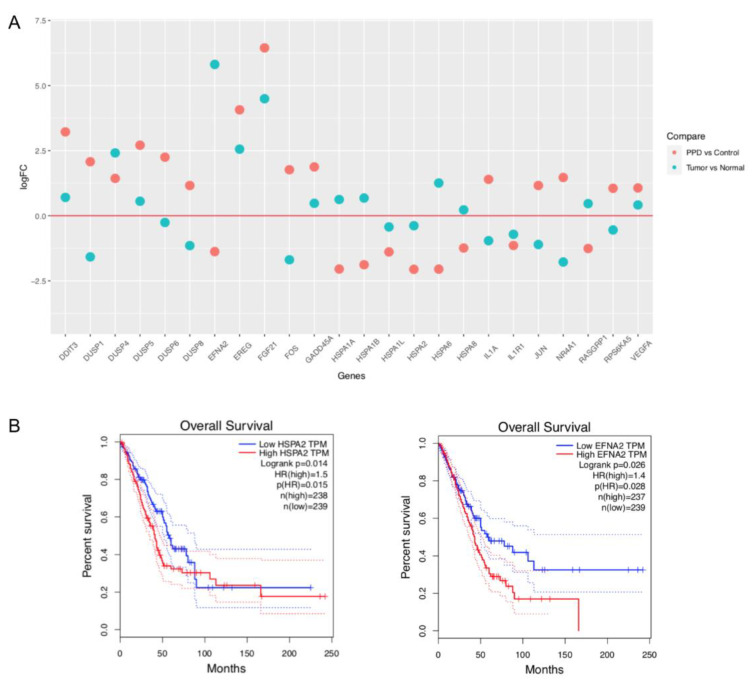
Comparison of PPD-regulated DEGs with clinical data of lung adenocarcinoma patients. (**A**): Dot plot of mRNA expression of PPD-regulated DEGs in lung adenocarcinoma patients. The data of mRNA expression profiles of lung adenocarcinoma were downloaded from The Cancer Genome Atlas (TCGA) website, and the log_2_-fold changes (tumors vs. normal tissues) of gene expression were calculated through R package EdgeR [28]. (**B**): Survival plots of DEGs *HSPA2* and *EFNA2* in lung adenocarcinoma patients (through the website of Gene Expression Profiling Interactive Analysis).

The data of mRNA expression profiles of lung adenocarcinoma (Table S7) were downloaded from The Cancer Genome Atlas (TCGA) website, and the log_2_-fold changes (tumors vs. normal tissues) of gene expression of the above DEGs enriched in the MAPK signaling pathway were calculated through R package EdgeR [28] (Table S8). As shown in Figure 4A, most downregulated genes in tumor tissues were upregulated in PPD-treated cells or vice versa indicating that PPD might reverse-regulate some genes that were upregulated or downregulated in tumors. We further investigated the association between the DEGs shown in Figure 4A and the overall survival of lung adenocarcinoma patients through the Gene Expression Profiling Interactive Analysis (GEPIA) website. Two PPD-downregulated genes *HSPA2* and *EFNA2* were screened out, which were significantly associated with patients’ overall survival (logrank *p* < 0.05, Figure 4B). Patients with low expression of the genes had better outcomes.

## 3. Discussion

Lung cancer is a serious threat to human health, and NSCLC is the main type of lung cancer [29]. In recent years, targeted drugs represented by epidermal growth factor receptor tyrosine kinase inhibitors (EGFR-TKI), such as gefitinib and erlotinib, have shown inspiring efficacy in the treatment of locally advanced and metastatic NSCLC, which specifically inhibit tumor cells with low side effects [30]. However, these drugs are primarily effective in patients with specific gene mutations in the EGFR, which limits their broad applications [11,12]. In the present study, we compared the effect of PPD with widely studied protopanaxadiol type ginsenosides Rc, Rg3, and Rh2 on EGFR wildtype human NSCLC NCI-H1299 cells. The results revealed that PPD has a stronger inhibitory effect on the cells than the above ginsenosides (PPD > Rh2 > Rg3 and Rc). In chemical structure, the three ginsenosides are all composed of 17 carbon dammarane skeletons. The difference lies in the different sugar moieties attached to the C-3 and C-20 positions (Figure S1). PPD is one of the major metabolites of protopanaxadiol-type ginsenosides following biodegradation, which has the same 17-carbon dammarane skeleton but no sugar moieties at the C-3 or C-20 positions. The differences in the chemical structure of ginsenosides might lead to different inhibitory effects on tumor cells, and the reduction of sugar chains may be beneficial for their anti-tumor activities [20].

Transcriptome sequencing analysis could reflect the changes in the transcription of the whole genome of cells following drug treatment. By analyzing the signaling pathways or biological processes the DEGs are involved in, it is possible to further speculate on the mechanism of action. Our results showed that 938 genes were upregulated and 466 genes were downregulated in PPD-treated NCI-H1299 cells compared with the control. The MAPK signaling pathway was the top pathway in the KEGG pathway enrichment analysis. MAPKs are serine-threonine kinases widely existing in eukaryotic cells, which transform extracellular stimuli into extensive cellular responses. The MAPK signaling pathway transduces extracellular stimulus signals into the cell and nucleus through a cascade of tertiary kinases: Extracellular signaling → MAPK kinase kinase → MAPK kinase → MAPK [31]. The MAPK signaling pathway is highly conserved in revolution, and the mammalian MAPKs primarily include the ERK, JNK, and p38 subfamilies that form parallel signaling pathways [32]. When cells are subjected to different stimuli, different cascades of MAPK signaling pathways were activated. Activated MAPKs phosphorylate a series of substrate proteins including transcription factors regulating biological processes within cells [33]. The MAPK signaling pathway plays a crucial role in cell proliferation, growth, apoptosis, and other activities. Its abnormal or excessive activation is associated with the occurrence and development of various tumors [31,34]. According to our results, the DEGs enriched in the MAPK signaling pathway were primarily involved in the ERK, JNK, and p38 axes. We further detected the phosphorylation of the three MAPKs through Western blotting. The phosphorylation of ERK and p38 was significantly decreased in PPD-treated cells, while the phosphorylation of JNK did not change. Many of the cancer-associated mutations of components that participate in the ERK signaling pathway have been found [35]. The ERK signaling pathway plays an important role in several steps of tumor development including tumor invasion, the survival of cancer cells, and resistance to anticancer drugs, which is therefore considered a prominent therapeutic target for cancer [34]. To date, inhibitors targeting the ERK signaling pathway, such as trametinib, are approved to treat NSCLC or are under clinical trial for the treatment of NSCLC [36]. In the research of Greenberg AK et al., p38 was activated in all of the human NSCLC samples, which might play a role in malignant cell growth or transformation [37]. It was reported that the downregulation of the caspase recruitment domain containing protein 9 enhanced the abilities of proliferation, invasion, and migration in NSCLC cells via activated p38 MAPK signaling [38]. The activation of p38 MAPK also contributes to the resistance to cisplatin or the fibroblast growth factor receptor (FGFR) inhibitor in NSCLC cells [39,40]. Therefore, our results indicated that PPD has the potential to be developed as an anticancer drug targeting ERK and the p38 MAPK signaling pathway for monotherapy or combination with chemotherapy or with other targeted inhibitors in NSCLC treatment.

We also compared the PPD-regulated DEGs enriched in the MAPK signaling pathway with the clinical data of lung adenocarcinoma patients, finding that most downregulated genes in tumor tissues were upregulated in PPD-treated cells or vice versa. Based on this, PPD treatment might benefit lung adenocarcinoma patients. We further investigated the correlation between the DEGs and the prognosis of lung adenocarcinoma patients. The results demonstrated that PPD-downregulated genes *HSPA2* and *EFNA2* were significantly associated with patients’ overall survival, with low expression indicating good outcomes. The human *HSPA2* gene is a member of the heat shock protein A (HSPA) multi-gene family coding for heat shock proteins with a 70 kDa molecular weight. In humans, the *HSPA* family genes are either constitutively expressed and/or induced in response to various pathological conditions and environmental stress. Members of the HSPA families are considered to play important roles in cancer [41]. According to Scieglinska D.’s research, HSPA2 was expressed in the majority of tumor histotypes including skin cancer, breast cancer, lung cancer, colon cancer, testis cancer, and so on. In NSCLC patients, nuclear HSPA2 expression was associated with histology, tumor-node-metastasis staging, and prognosis. High HSPA2 expression was correlated with poor prognosis [42]. Pan-HSPA inhibitors showed a potent anticancer effect on NSCLC cells and sensitized NSCLC cells to bortezomib [43]. Ephrin-A2 (EFNA2) is one of the ephrin family ligands for receptor tyrosine kinase EPH family receptors, which are the target of the WNT/beta-catenin signaling pathway implicated in embryogenesis, tissue regeneration, and carcinogenesis [44]. The mRNA expression of EFNA2 was upregulated in most cancer types including lung adenocarcinoma, lung squamous cell carcinoma, breast invasive carcinoma, colon adenocarcinoma, gastric carcinoma, esophageal carcinoma, pancreatic adenocarcinoma, bladder urothelial carcinoma, prostate adenocarcinoma, ovarian serous cystadenocarcinoma, uterine corpus endometrial carcinoma, skin cutaneous melanoma, acute myeloid leukemia, and others [45,46]. Overexpression of EFNA2 in prostate cancer cells could accelerate cell migration and invasion in vitro and facilitate tumor metastasis and angiogenesis in xenograft mouse models while silencing of this gene reversed the above effects [47]. Synthesized EFNA2-targeted immunoliposomes showed significant antitumor activity in NSCLC and triple-negative breast cancer xenograft models. The lead molecule entered a Phase I clinical trial in patients with solid tumors [48]. To summarize, PPD inhibited human NSCLC NCI-H1299 cells by regulating the ERK and p38 MAPK signaling pathways and downregulated key genes correlated with poor prognosis in NSCLC patients. It has the potential to be developed as drugs for NSCLC treatment targeting ERK and p38 MAPK pathways.

## 4. Materials and Methods

### 4.1. Cell Line and Reagents

The human NSCLC cell line NCI-H1299 and human normal fibroblast cell line BJ were from the American Type Culture Collection and were conserved in our lab. The cells were maintained in the RPMI-1640 medium supplemented with 10% fetal bovine serum (Sigma-Aldrich, Shanghai, China) and incubated in a 5% CO_2_ humidified incubator at 37 °C. The cells were digested with 0.25% trypsin (Sigma-Aldrich, Shanghai, China) for subculture. All experiments were performed during the logarithmic growth phase of cells. 20(S)-PPD and ginsenosides 20(S)-Rh2, 20(S)-Rg3, and 20(S)-Rc were purchased from Shanghai Yuanye Biotechnology Co., Ltd. (Shanghai, China) and dissolved in dimethyl sulfoxide (DMSO) at a concentration of 10 mg/mL. All experiments were performed at Jilin University, Changchun, China. This study was supported by the Department of Science and Technology of Jilin Province, China (No. 20200201393JC, 8 June 2020).

### 4.2. Cell Viability Detection

NCI-H1299 cells were seeded into a 96-well plate with 3000 cells per well in 100 μL of complete medium for 12 h. Then ginsenosides or PPD diluted into different concentrations of 100 μL of the complete medium were added to each well. Additional DMSO was added to wells to ensure that all the wells had the same concentration of DMSO. Cell viability was detected using CCK-8 [49]. After continuing to cultivate for 24, 48, and 72 h, the culture medium was discarded, and 100 μL of the complete medium containing 10% of CCK-8 (MedChemExpress, Shanghai, China) solution was added to each well. After continuing to cultivate for 1–3 h, the absorbance (A) at 450 nm was measured using a microplate ELISA reader (Perlong, Beijing, China). The inhibition of cell growth was calculated as:

Inhibition rate = [(A_cell control_ − A_sample_)/(A_cell control_ − A_blank_)] × 100%. (1)

The half-maximal inhibitory concentration (IC_50_) was calculated using GraphPad Prism version 8.4.3 software. All of these experiments were performed in triplicate.

### 4.3. Cell Apoptosis and Cell Cycle Analyses

NCI-H1299 cells were seeded into a 6-well plate with 5 × 10^5^ cells per well in 3 mL of complete medium for 12 h. Then PPD (at a final concentration of 26 μg/mL) or an equal amount of DMSO was added. After continuing to cultivate for 48 h, the cells were harvested, and the cell apoptosis, cell mitochondrial membrane potential, and cell cycle were detected using the Annexin V-FITC Apoptosis Detection Kit (Beyotime, Shanghai, China) [50], the enhanced mitochondrial membrane potential detection kit (JC-1, Beyotime, Shanghai, China) [51], and the Cell Cycle and Apoptosis Analysis Kit (Beyotime, Shanghai, China) [52], respectively. To detect cell apoptosis, the cells were digested with 0.25% trypsin, then washed twice with PBS and resuspended in 500 μL of the binding buffer. Then 5 μL of the Annexin V-FITC solution was added and protected from light at room temperature for 15 min, and 3 μL of the PI solution was added and protected from light at room temperature for 5 min. The cells were centrifuged and resuspended in 400 μL of binding buffer before being analyzed by a flow cytometer. For cell mitochondrial membrane potential analysis, the cells were digested, centrifuged, and resuspended in 0.5 mL of the complete medium. Then, 0.5 mL of the JC-1 staining solution was added, mixed gently, and incubated at 37 °C for 20 min. After being washed twice with JC-1 staining buffer, the cells were resuspended with 0.5 mL of JC-1 staining buffer before flow cytometry analysis. To detect the cell cycle, the digested cells were washed twice with PBS and fixed at 4 °C overnight with ice-cold 70% ethanol. Then the cells were centrifuged, washed twice with PBS, and resuspended in 0.5 mL of PI staining buffer. After being treated with RNase for 30 min at 37 °C in darkness, the cells were analyzed by flow cytometry. For analyzing cell apoptosis, cell mitochondrial membrane potential, and the cell cycle, standard flow cytometry procedures were used (BD FACSCalibur Flow Cytometer, BD Biosciences, San Jose, CA, USA). The data were processed by FlowJo 10.8.1 software.

### 4.4. RNA Sequencing

NCI-H1299 cells were seeded into a 6-cm plate with 2 × 10^5^ cells per well. After adherence, the cells were treated with 26 μg/mL of PPD or an equal amount of DMSO for another 48 h. Then the cells were washed twice with PBS, lysed, and preserved in 1 mL of TRIzol reagent (Invitrogen, Shanghai, China). RNA purification, library preparation, and RNA sequencing were completed by GENEWIZ Co., Ltd. (Suzhou, China) [53,54]. Briefly, total RNA was extracted with TRIzol reagent, and RNA integrity was qualified by the Agilent 2100 Bioanalyzer (Agilent Technologies, Palo Alto, CA, USA), NanoDrop (Thermo Fisher Scientific Inc., Shanghai, China), and 1% agrose gel. Poly(A) mRNA isolation was performed using the NEBNext Poly(A) mRNA Magnetic Isolation Module (NEB), libraries were constructed using the NEBNext^®^ UltraTM RNA Library Prep Kit for Illumina^®^ (NEB, Ipswich, MA, USA), and library preparations were sequenced on the Illumina HiSeq X Ten platform. Sequencing was carried out using a 2 × 150 bp paired-end configuration.

### 4.5. Analysis of RNA Sequencing Data

R software version 4.3.1 and selected packages were used to analyze the data of RNA sequencing (http://www.r-project.org, accessed on 30 August 2022). The quality of reads was evaluated through FastQC (https://www.bioinformatics.babraham.ac.uk/projects/fastqc/, accessed on 2 September 2022). The quality of bases lower than 33, adapters, and other technical sequences was removed by Cutadapt (version 1.9.1, https://cutadapt.readthedocs.io/en/stable/installation.html, accessed on 6 September 2022). Clean reads were mapped onto the Ensembl human reference genome (EnsemblGRCh37 release 98) through aligner software Hisat2 (v2.0.1) [55]. The R package EdgeR [28] was used to analyze the DEGs, and the R package pheatmap [56] was used to generate a heatmap base on the FPKM of each group. The R package clusterProfiler [57,58] was used to conduct the KEGG pathway enrichment analysis of DEGs. The map of the MAPK signaling pathway was downloaded from the KEGG website (https://www.kegg.jp/pathway/map04010, accessed on 9 September 2022), and the gene expression changes enriched in this pathway were integrated and visualized on this map through the R package Pathview [59].

### 4.6. Western Blotting

For the Western blotting assay [60], NCI-H1299 cells were seeded into a 6-cm plate with 2 × 10^5^ cells per well. After adherence, the cells were treated with 26 μg/mL of PPD or an equal amount of DMSO for another 48 h. The cells were digested with trypsin and washed with PBS. RIPA lysis buffer (P0013D, Beyotime, Shanghai, China) was used to extract the total protein of cells. The proteins were separated by SDS-PAGE. The proteins were then transferred to a PVDF membrane (Thermo Fisher Scientific Inc., Shanghai, China) and blocked with 5% non-fat milk followed by incubation with primary antibodies. The antibodies were purchased from Cell Signaling Technology (CST, Shanghai, China) including p44/42 MAPK (Erk1/2) (137F5) rabbit mAb (#4695, 1:1000), SAPK/JNK rabbit mAb (#9252, 1:1000), or p38 MAPK (D13E1) XP^®^ rabbit mAb (#8690, 1:1000). The Histone H3 Antibody (#AF0863, Affinity Biosciences, Changzhou, China, 1:2000) was used for loading control. Then an anti-rabbit IgG HRP-linked antibody (#7074, CST, 1:2000) was added, and the signals were detected using the super ECL detection reagent (Yeasen, Shanghai, China). For the detection of phospho-proteins, the PVDF membrane was treated with stripping buffer (Beyotime, Shanghai, China) to remove the binding antibodies followed by incubation with phospho-p44/42 MAPK (Erk1/2) (Thr202/Tyr204) (D13.14.4E) XP^®^ rabbit mAb (#4370, CST, 1:2000), phospho-SAPK/JNK (Thr183/Tyr185) (81E11) rabbit mAb (#4668, CST, 1:1000), or phospho-p38 MAPK (Thr180/Tyr182) (D3F9) XP^®^ rabbit mAb (#4511, CST, 1:1000). The steps for incubating with the secondary antibody and detecting signals were the same as above.

### 4.7. Lung Adenocarcinoma Data Acquisition and Analysis

The mRNA expression profiles data of human lung adenocarcinoma were downloaded from the website of TCGA (https://portal.gdc.cancer.gov/, accessed on 27 April 2023) through the R package TCGAbiolinks [61] and processed through the R package tidyverse [62]. The data contained the mRNA expression profiles and clinical information of patients of a total of 598 samples from lung adenocarcinoma patients, including 539 samples from tumor tissues and 59 samples from adjacent normal tissues. The R package EdgeR [28] was used to investigate the log_2_-fold change of gene expression between tumors and normal tissues with a focus on the DEGs enriched in the MAPK signaling pathway. Survival plots of DEGs in lung adenocarcinoma patients were generated using the GEPIA website (http://gepia.cancer-pku.cn/, accessed on 28 April 2023) setting the group cutoff as the median.

### 4.8. Statistical Analysis

All data were collected from at least triplicate tests and were displayed as mean ± standard deviation (mean ± SD). Statistical analysis was performed with GraphPad Prism version 8.4.3 software. A double-tailed Student’s *t*-test was used to analyze the differences between the two groups. *p* < 0.05 was considered to be statistically significant.

## 5. Conclusions

PPD, as a major metabolite of protopanaxadiol type ginsenosides, has a stronger inhibitory effect on NCI-H1299 human NSCLC cells than ginsenosides Rh2, Rg3, and Rc. It could inhibit proliferation and induce apoptosis in NCI-H1299 cells by affecting gene expression and regulating the ERK and p38 MAPK signaling pathways. Among the PPD-regulated DEGs enriched in the MAPK signaling pathway, most downregulated genes in human lung adenocarcinoma tissues were upregulated in PPD-treated cells or vice versa. Moreover, two PPD-downregulated genes, *HSPA2* and *EFNA2,* were associated with patients’ overall survival. Therefore, PPD has the potential to be developed as a drug targeting ERK and p38 MAPK pathways, which might benefit NSCLC patients in monotherapy or in combination with chemotherapy or other targeted inhibitor treatments.

## Data Availability

The raw data of RNA-seq were submitted to the Sequence Read Archive (SAR) of National Center for Biotechnology Information (https://www.ncbi.nlm.nih.gov/bioproject, accessed on 21 May 2023). BioProject ID PRJNA977110 (the data will be released after the manuscript is published).

## Supplementary Materials

The following supporting information can be downloaded at: https://www.mdpi.com/article/10.3390/molecules28155746/s1, Figure S1: Molecular structures of PPD and ginsenosides Rc, Rg3, and Rh2; Figure S2: Dose effects of PPD and ginsenoside Rh2 on NCI-H1299 cells; Figure S3: Effects of PPD on cell cycle of NCI-H1299 cells; Figure S4: RNA analysis; Figure S5: Sequencing depth of RNA sequencing; Table S1: FPKM and Read counts of each transcript; Table S2: Gene expression changes of PPD vs. Control; Table S3: Differentially expressed genes; Table S4: Enriched KEGG pathways of DEGs; Table S5: DEGs enriched in MAPK pathway; Table S6: Expression changed genes in MAPK pathway; Table S7: mRNA expression profiles of lung adenocarcinoma; Table S8: DEGs of lung adenocarcinoma.

## Sample Availability

Samples of the compounds are available from the authors.

## Abbreviations

A	absorbance
CC_50_	50% cytotoxic concentration
CCK-8	cell counting kit-8
DEG	differentially expressed gene
DMSO	dimethyl sulfoxide
EFNA2	Ephrin-A2
EGFR-TKI	epidermal growth factor receptor tyrosine kinase inhibitors
ELISA	enzyme-linked immunosorbent assay
ERK	extracellular signal-regulated kinase
FGFR	fibroblast growth factor receptor
FITC	fluorescein isothiocyanate
FPKM	fragments per kilobase million
GEPIA	Gene Expression Profiling Interactive Analysis
HSPA	heat shock protein A
IC_50_	half maximal inhibitory concentration
JNK	c-Jun NH_2_-terminal kinase
KEGG	Kyoto Encyclopedia of Genes and Genomes
MAPK	mitogen-activated protein kinases
NSCLC	non-small cell lung cancer
PI	propidium iodide
PPD	20(S)-Protopanaxadiol
RIN	RNA integrity
SD	standard deviation
TCGA	The Cancer Genome Atlas
