# Supplementary material for: Transcriptome Analysis of the Inhibitory Effects of 20(S)-Protopanaxadiol on NCI-H1299 Non-Small Cell Lung Cancer Cells
Source: Molecules. 2023 Jul 29;28(15):5746. doi: 10.3390/molecules28155746 (PMC10421167; doi:10.3390/molecules28155746)
Supplement: Supplementary file 1 [file molecules-28-05746-s001.zip › Figure S1 Molecular structures.pdf]

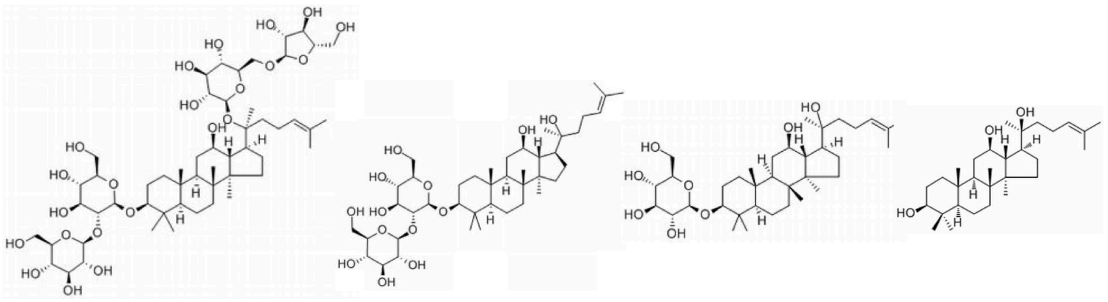

Rc Rg3 Rh2 PPD

Figure S1. Molecular structures of PPD and ginsenosides Rc, Rg3 and Rh2. The molecular structure graphs were downloaded from the website of Shanghai Yuanye Biotechnology Co., Ltd (https://www.shyuanye.com/, accessed on 5 May 2023).
