# Supplementary material for: Transcriptome Analysis of the Inhibitory Effects of 20(S)-Protopanaxadiol on NCI-H1299 Non-Small Cell Lung Cancer Cells
Source: Molecules. 2023 Jul 29;28(15):5746. doi: 10.3390/molecules28155746 (PMC10421167; doi:10.3390/molecules28155746)
Supplement: Supplementary file 1 [file molecules-28-05746-s001.zip › Figure S2.pdf]

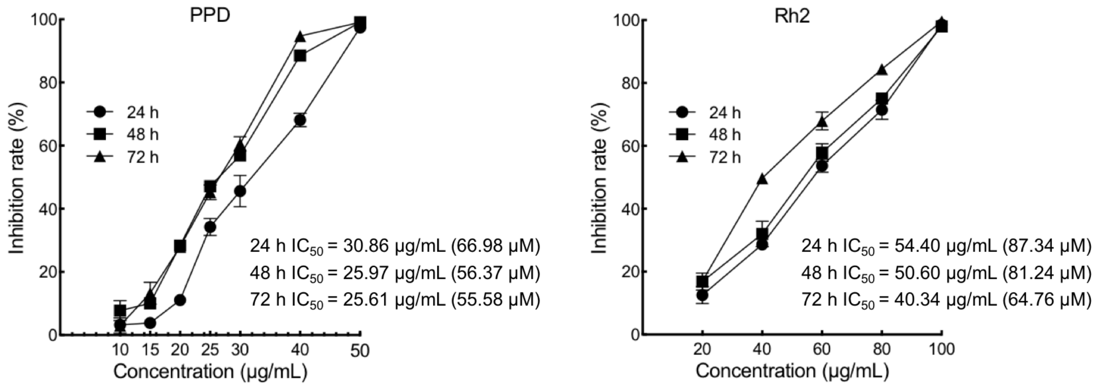

Figure S2. Dose-effects of PPD and ginsenoside Rh2 on NCI-H1299 cells. Cell viability was detected through CCK-8 assay after cells were treated with PPD (10, 15, 20, 25, 30, 40 and 50 μg/mL) and Rh2 (20, 40, 60, 80 and 100 μg/mL) for 24, 48 and 72 h.
