# Supplementary material for: Transcriptome Analysis of the Inhibitory Effects of 20(S)-Protopanaxadiol on NCI-H1299 Non-Small Cell Lung Cancer Cells
Source: Molecules. 2023 Jul 29;28(15):5746. doi: 10.3390/molecules28155746 (PMC10421167; doi:10.3390/molecules28155746)
Supplement: Supplementary file 1 [file molecules-28-05746-s001.zip › Figure S3.pdf]

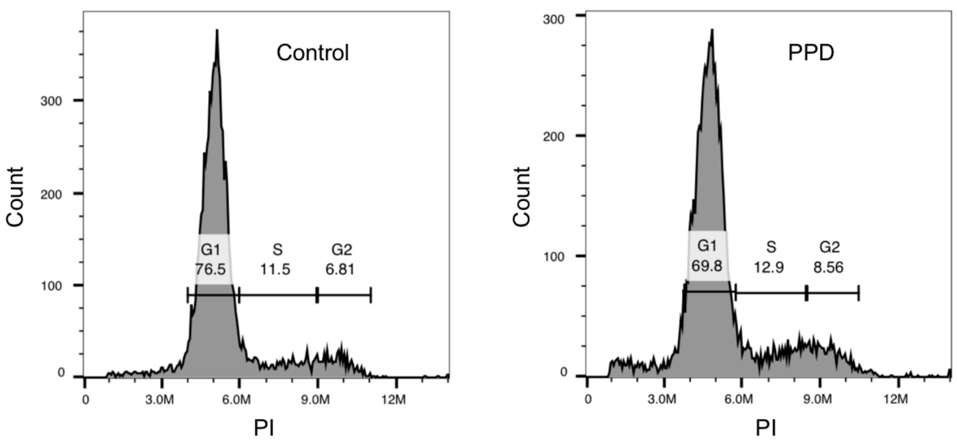

Figure S3. Effects of PPD on cell cycle of NCI-H1299 cells. Cell cycle was investigated through flow cytometry after PPD (26 µg/mL) treatment for 48 h.
