# Supplementary material for: Transcriptome Analysis of the Inhibitory Effects of 20(S)-Protopanaxadiol on NCI-H1299 Non-Small Cell Lung Cancer Cells
Source: Molecules. 2023 Jul 29;28(15):5746. doi: 10.3390/molecules28155746 (PMC10421167; doi:10.3390/molecules28155746)
Supplement: Supplementary file 1 [file molecules-28-05746-s001.zip › Figure S4 RNA analysis.pdf]

Filename: N1900946-1-9-20190219-2200.RNA

Gel Images

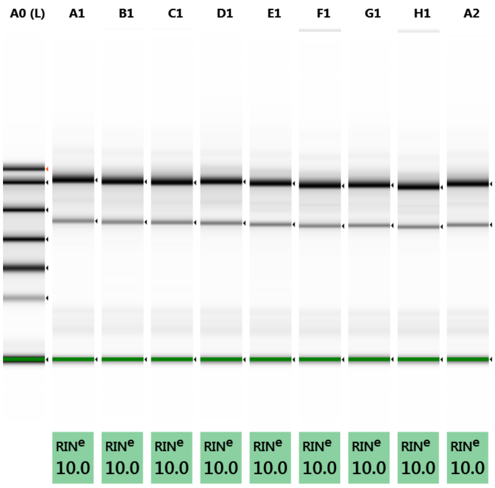

D1: 20(S)-PPD-1
E1: 20(S)-PPD-2
F1: 20(S)-PPD-3
G1: Control-1
H1: Control-2
A2: Control-3

Default image (Contrast 50%), Image is Scaled to Sample, Image is Scaled to view larger Molecular Weight range

Sample Info

Well	RINe	28S/18S (Area)	Conc. [ng/µl]	Sample Description	Alert	Observations
A0	-	-	97.5	Electronic Ladder		Ladder
A1	10.0	2.9	124	R1 25XDilution		
B1	10.0	2.8	129	R2 19XDilution		
C1	10.0	3.1	129	R3 20XDilution		
D1	10.0	2.8	112	S1 28XDilution		
E1	10.0	2.8	103	S2 17XDilution		
F1	10.0	2.9	120	S3 21XDilution		
G1	10.0	3.0	97.4	CON-1 21XDilution		
H1	10.0	3.0	116	CON-2 22XDilution		
A2	10.0	3.4	94.3	CON-3 11XDilution		

 © Agilent Technologies, Inc. 2016

A0: Electronic Ladder

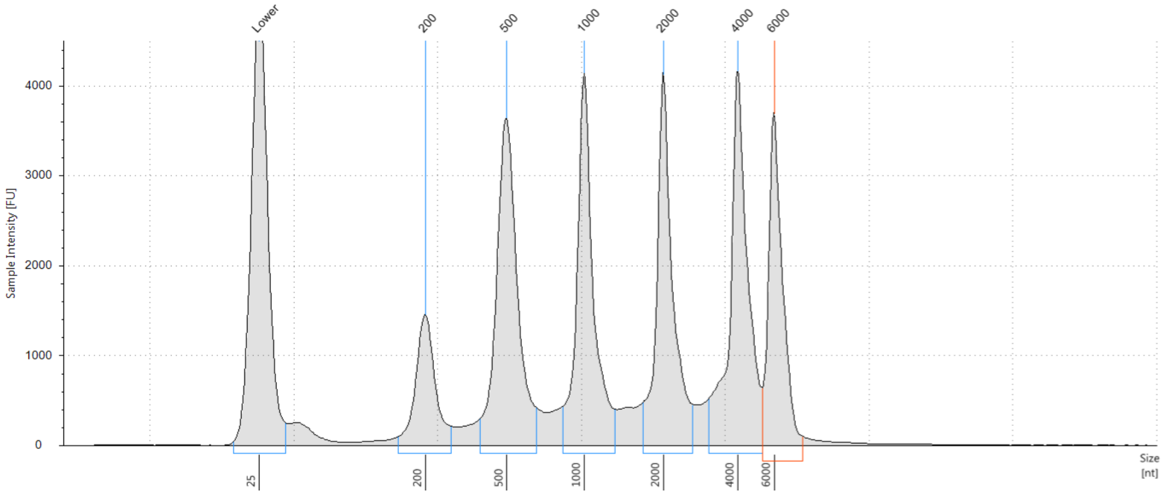

Sample Table

Well	RINe	28S/18S (Area)	Conc. [ng/µl]	Sample Description	Alert	Observations
A0	-	-	97.5	Electronic Ladder		Ladder

Peak Table

Size [nt]	Calibrated Conc. [ng/µl]	Assigned Conc. [ng/µl]	Peak Molarity [nmol/l]	% Integrated Area	Peak Comment	Observations
25	40.0	40.0	4710	-		Lower Marker
200	6.82	-	100	7.80		
500	18.3	-	107	20.88		
1000	16.3	-	47.9	18.62		
2000	15.8	-	23.3	18.11		
4000	17.8	-	13.1	20.38		
6000	12.4	-	6.10	14.22		

© Agilent Technologies, Inc. 2016

A1: R1 25XDilution

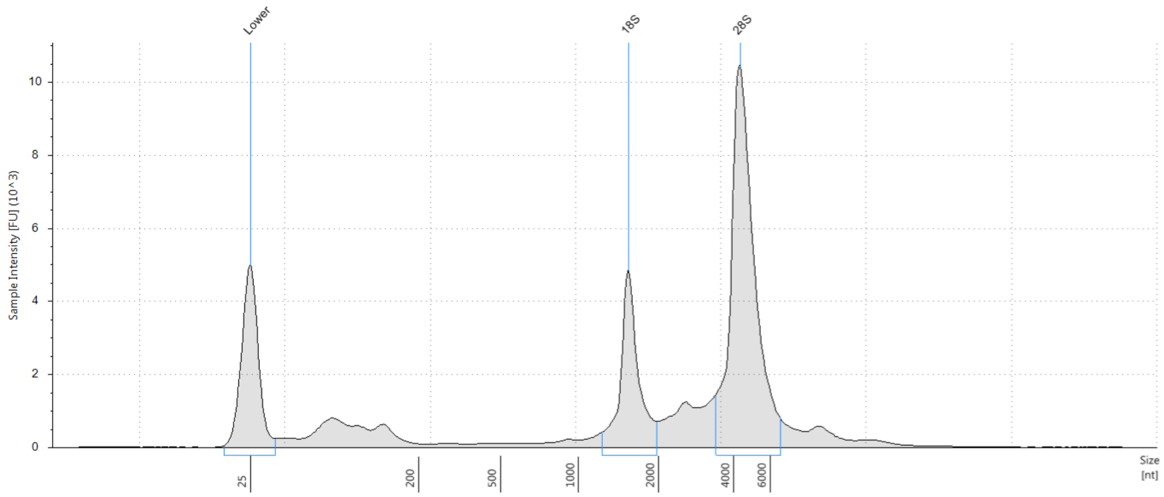

Sample Table

Well	RINe	28S/18S (Area)	Conc. [ng/µl]	Sample Description	Alert	Observations
A1	10.0	2.9	124	R1 25XDilution		

Peak Table

Size [nt]	Calibrated Conc. [ng/µl]	Assigned Conc. [ng/µl]	Peak Molarity [nmol/l]	% Integrated Area	Peak Comment	Observations
25	40.0	40.0	4710	-		Lower Marker
1532	21.2	-	40.8	25.62		18S
4266	61.7	-	42.5	74.38		28S

B1: R2 19XDilution

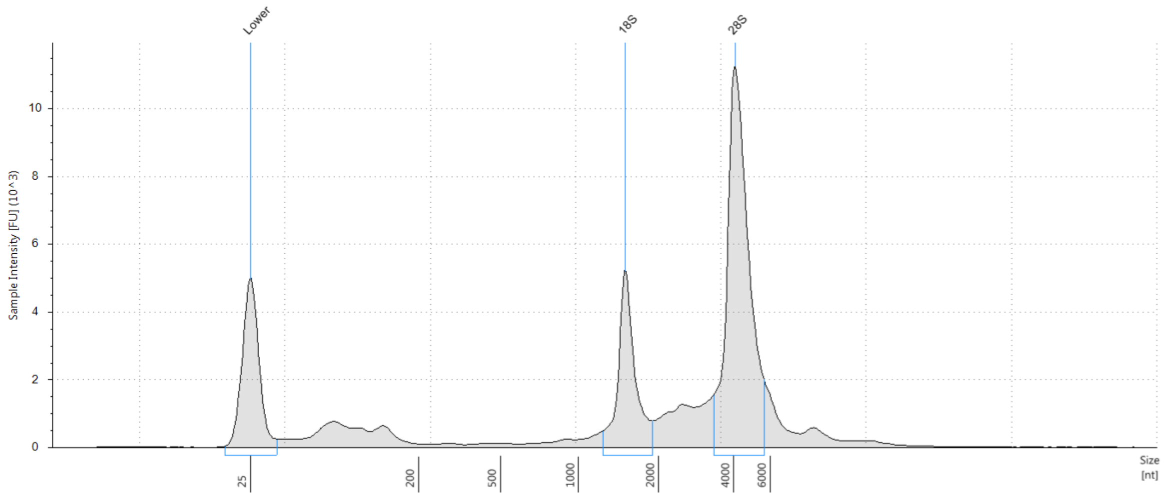

Sample Table

Well	RINe	28S/18S (Area)	Conc. [ng/µl]	Sample Description	Alert	Observations
B1	10.0	2.8	129	R2 19XDilution		

Peak Table

Size [nt]	Calibrated Conc. [ng/µl]	Assigned Conc. [ng/µl]	Peak Molarity [nmol/l]	% Integrated Area	Peak Comment	Observations
25	40.0	40.0	4710	-		Lower Marker
1497	21.6	-	42.4	26.10		18S
4065	61.2	-	44.2	73.90		28S

C1: R3 20XDilution

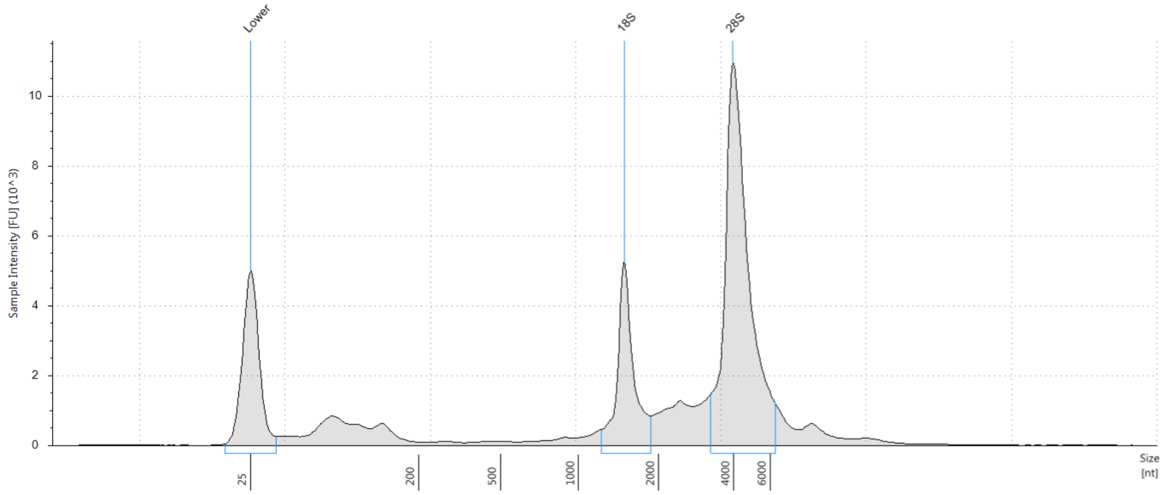

Sample Table

Well	RINe	28S/18S (Area)	Conc. [ng/µl]	Sample Description	Alert	Observations
C1	10.0	3.1	129	R3 20XDilution		

Peak Table

Size [nt]	Calibrated Conc. [ng/µl]	Assigned Conc. [ng/µl]	Peak Molarity [nmol/l]	% Integrated Area	Peak Comment	Observations
25	40.0	40.0	4710	-		Lower Marker
1479	20.7	-	41.2	24.10		18S
3954	65.3	-	48.5	75.90		28S

 © Agilent Technologies, Inc. 2016

D1: S1 28XDilution

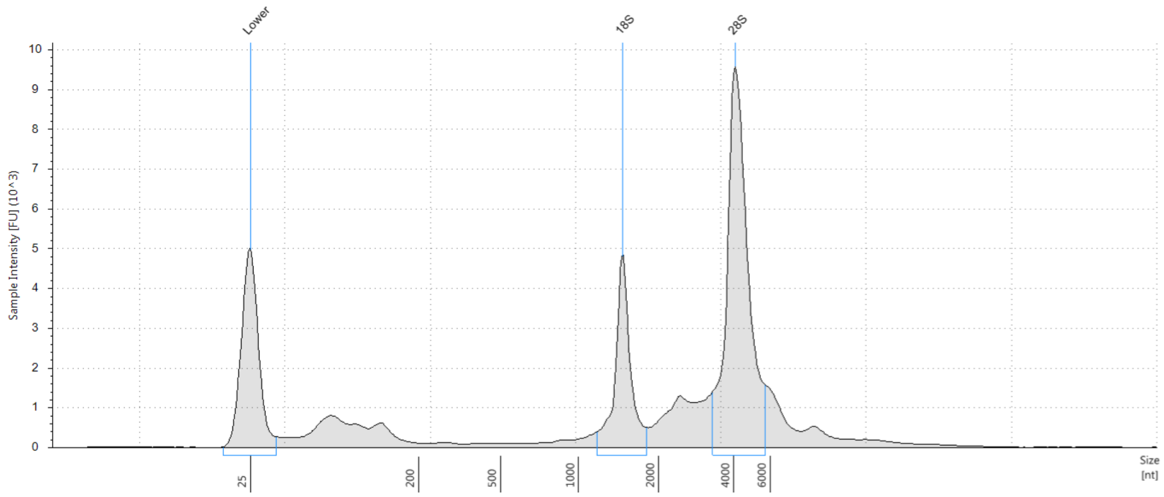

Sample Table

Well	RINe	28S/18S (Area)	Conc. [ng/µl]	Sample Description	Alert	Observations
D1	10.0	2.8	112	S1 28XDilution		

Peak Table

Size [nt]	Calibrated Conc. [ng/µl]	Assigned Conc. [ng/µl]	Peak Molarity [nmol/l]	% Integrated Area	Peak Comment	Observations
25	40.0	40.0	4710	-		Lower Marker
1462	17.6	-	35.4	26.28		18S
4062	49.3	-	35.7	73.72		28S

 © Agilent Technologies, Inc. 2016

E1: S2 17XDilution

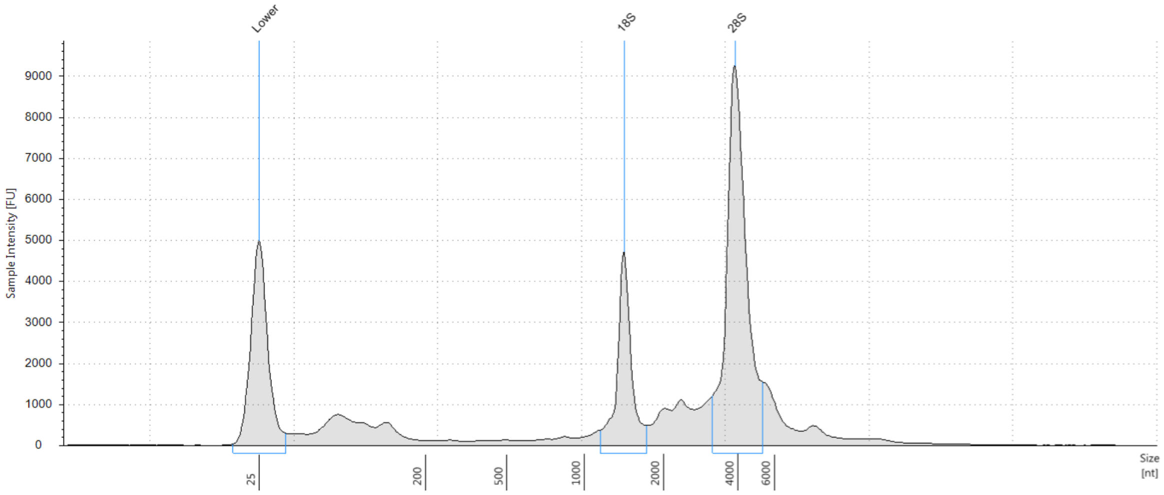

Sample Table

Well	RINe	28S/18S (Area)	Conc. [ng/µl]	Sample Description	Alert	Observations
E1	10.0	2.8	103	S2 17XDilution		

Peak Table

Size [nt]	Calibrated Conc. [ng/µl]	Assigned Conc. [ng/µl]	Peak Molarity [nmol/l]	% Integrated Area	Peak Comment	Observations
25	40.0	40.0	4710	-		Lower Marker
1420	15.9	-	33.0	25.98		18S
3890	45.4	-	34.3	74.02		28S

 © Agilent Technologies, Inc. 2016

F1: S3 21XDilution

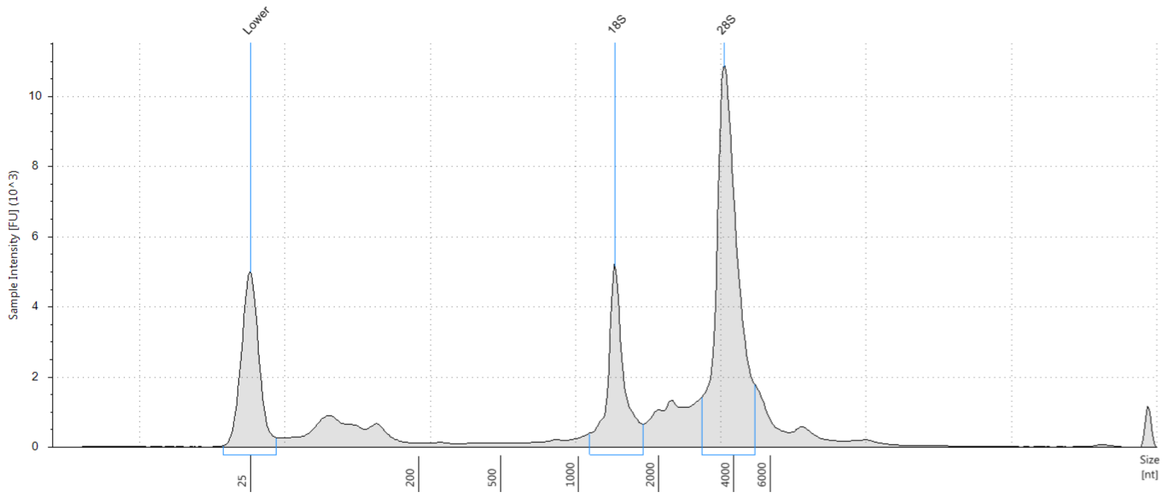

Sample Table

Well	RINe	28S/18S (Area)	Conc. [ng/µl]	Sample Description	Alert	Observations
F1	10.0	2.9	120	S3 21XDilution		

Peak Table

Size [nt]	Calibrated Conc. [ng/µl]	Assigned Conc. [ng/µl]	Peak Molarity [nmol/l]	% Integrated Area	Peak Comment	Observations
25	40.0	40.0	4710	-		Lower Marker
1367	19.1	-	41.1	25.51		18S
3657	55.8	-	44.9	74.49		28S

© Agilent Technologies, Inc. 2016

G1: CON-1 21XDilution

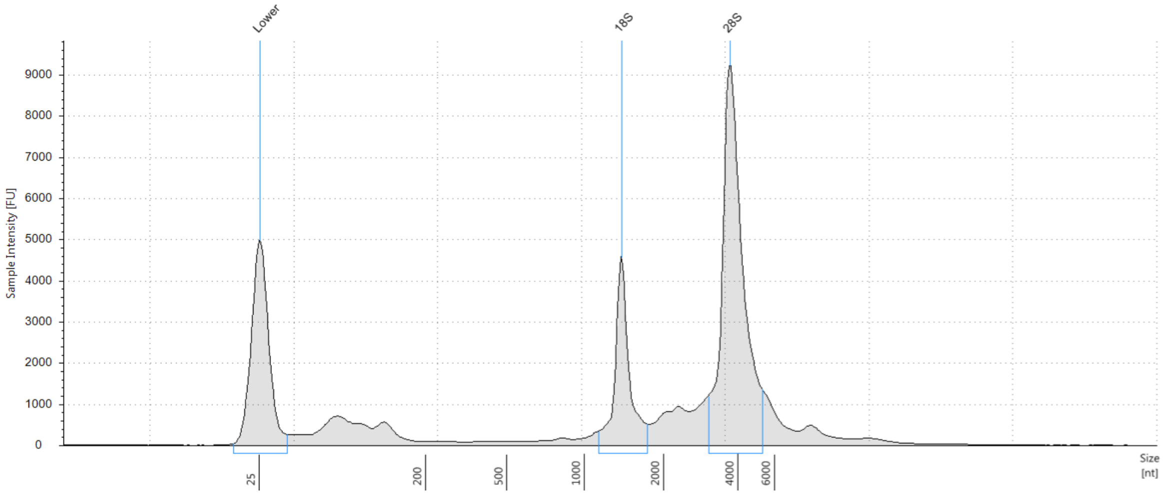

Sample Table

Well	RINe	28S/18S (Area)	Conc. [ng/µl]	Sample Description	Alert	Observations
G1	10.0	3.0	97.4	CON-1 21XDilution		

Peak Table

Size [nt]	Calibrated Conc. [ng/µl]	Assigned Conc. [ng/µl]	Peak Molarity [nmol/l]	% Integrated Area	Peak Comment	Observations
25	40.0	40.0	4710	-		Lower Marker
1375	15.3	-	32.7	24.91		18S
3695	46.1	-	36.7	75.09		28S

 © Agilent Technologies, Inc. 2016

H1: CON-2 22XDilution

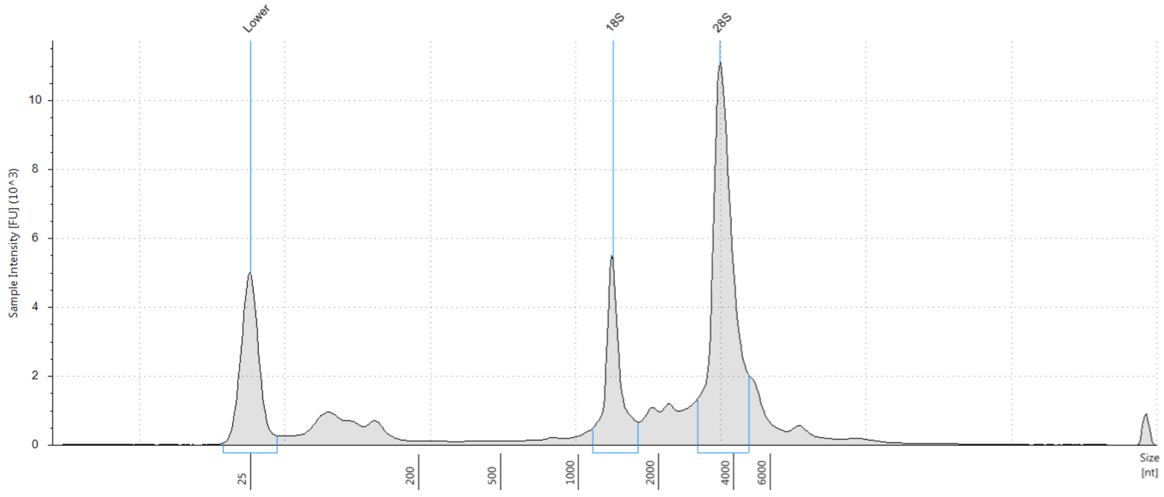

Sample Table

Well	RINe	28S/18S (Area)	Conc. [ng/µl]	Sample Description	Alert	Observations
H1	10.0	3.0	116	CON-2 22XDilution		

Peak Table

Size [nt]	Calibrated Conc. [ng/µl]	Assigned Conc. [ng/µl]	Peak Molarity [nmol/l]	% Integrated Area	Peak Comment	Observations
25	40.0	40.0	4710	-		Lower Marker
1341	17.8	-	39.0	25.06		18S
3509	53.1	-	44.5	74.94		28S

 © Agilent Technologies, Inc. 2016

A2: CON-3 11XDilution

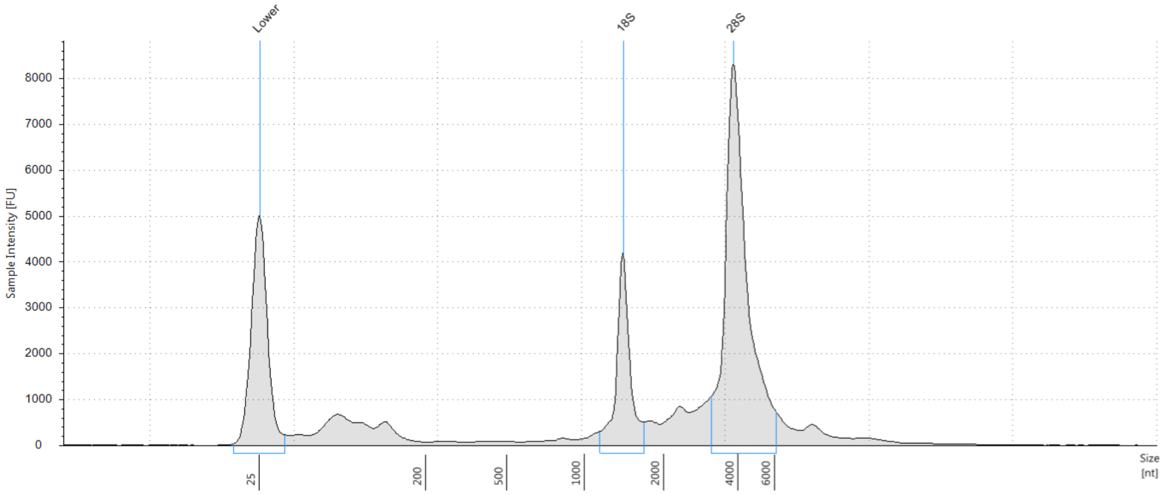

Sample Table

Well	RINe	28S/18S (Area)	Conc. [ng/µl]	Sample Description	Alert	Observations
A2	10.0	3.4	94.3	CON-3 11XDilution		

Peak Table

Size [nt]	Calibrated Conc. [ng/µl]	Assigned Conc. [ng/µl]	Peak Molarity [nmol/l]	% Integrated Area	Peak Comment	Observations
25	40.0	40.0	4710	-		Lower Marker
1402	14.1	-	29.6	22.90		18S
3825	47.5	-	36.5	77.10		28S

 © Agilent Technologies, Inc. 2016
