# Supplementary figures and images for: Transcriptome Analysis of the Inhibitory Effects of 20(S)-Protopanaxadiol on NCI-H1299 Non-Small Cell Lung Cancer Cells
Source: Molecules. 2023 Jul 29;28(15):5746. doi: 10.3390/molecules28155746 (PMC10421167; doi:10.3390/molecules28155746)

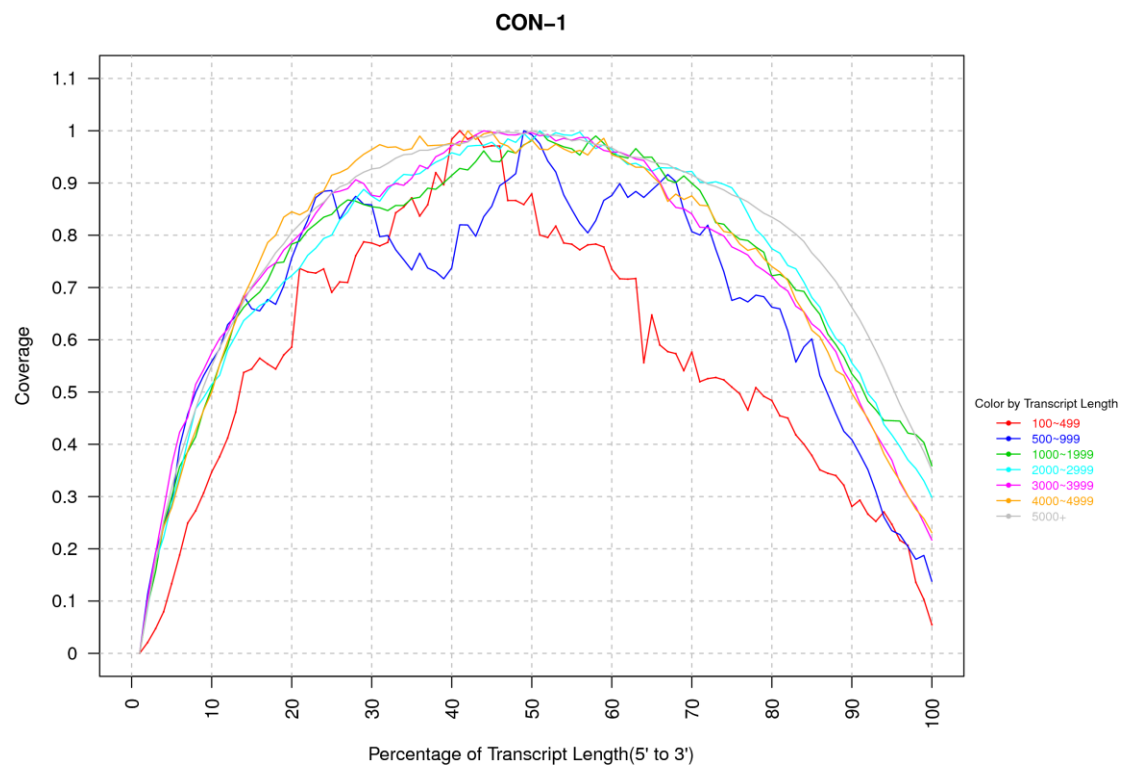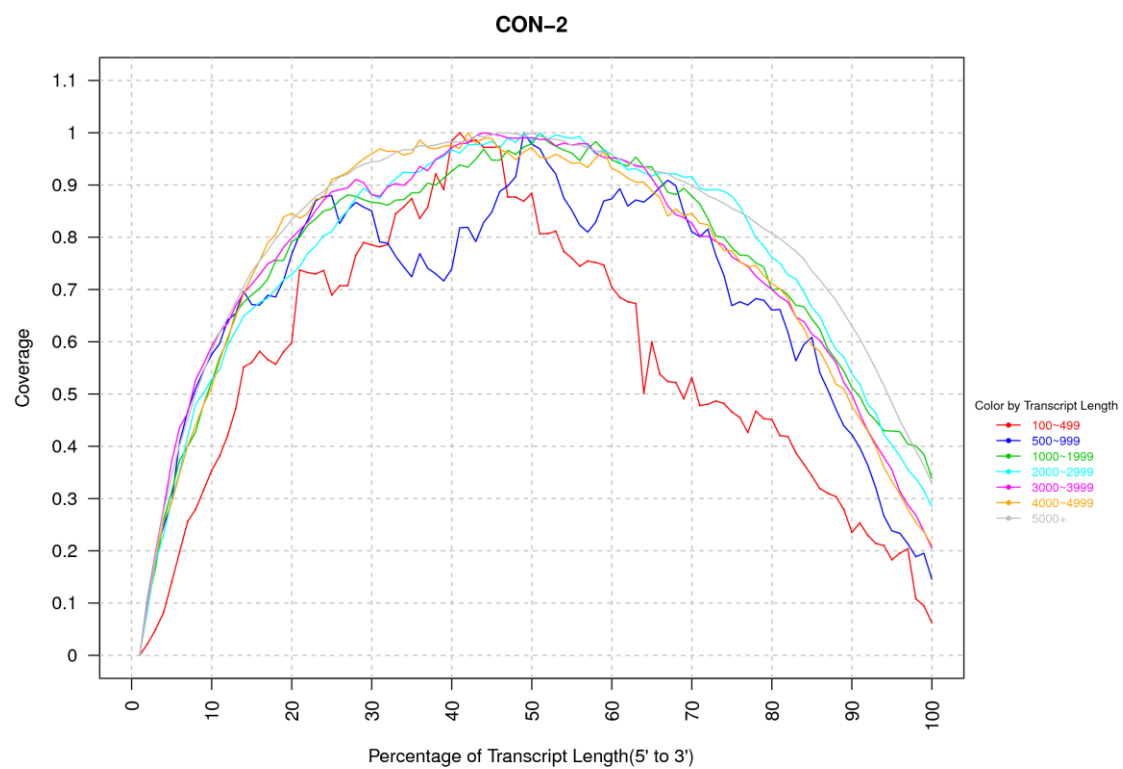

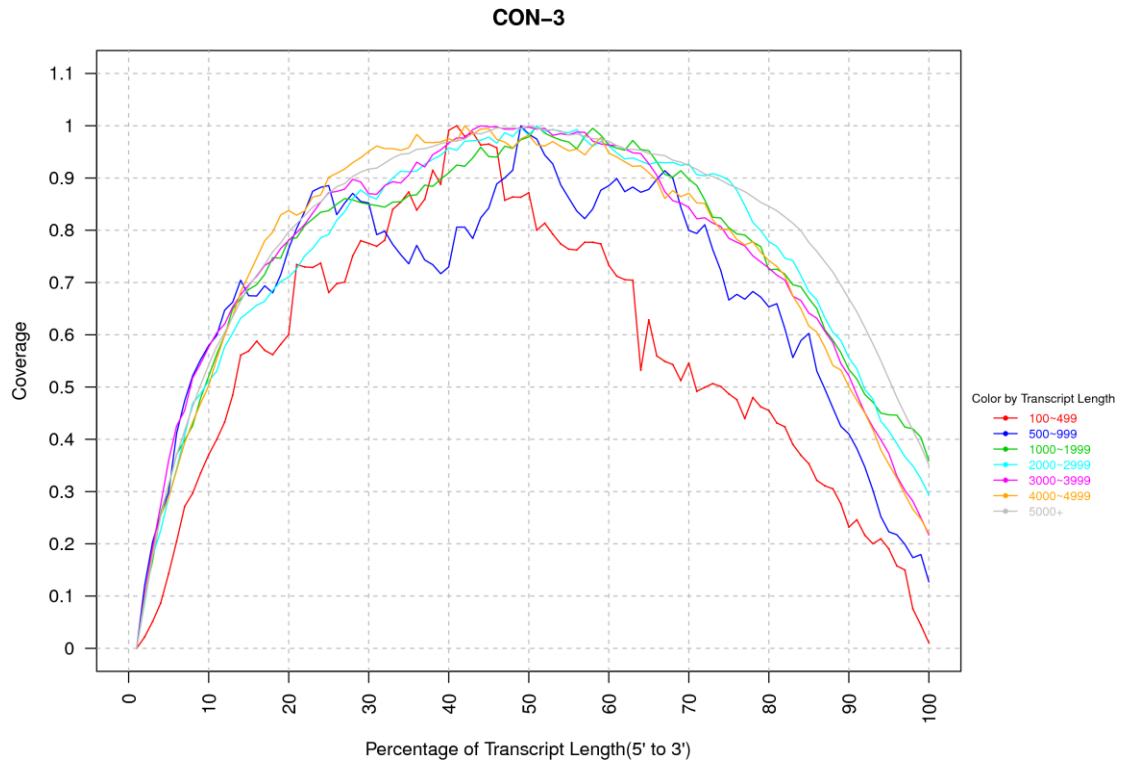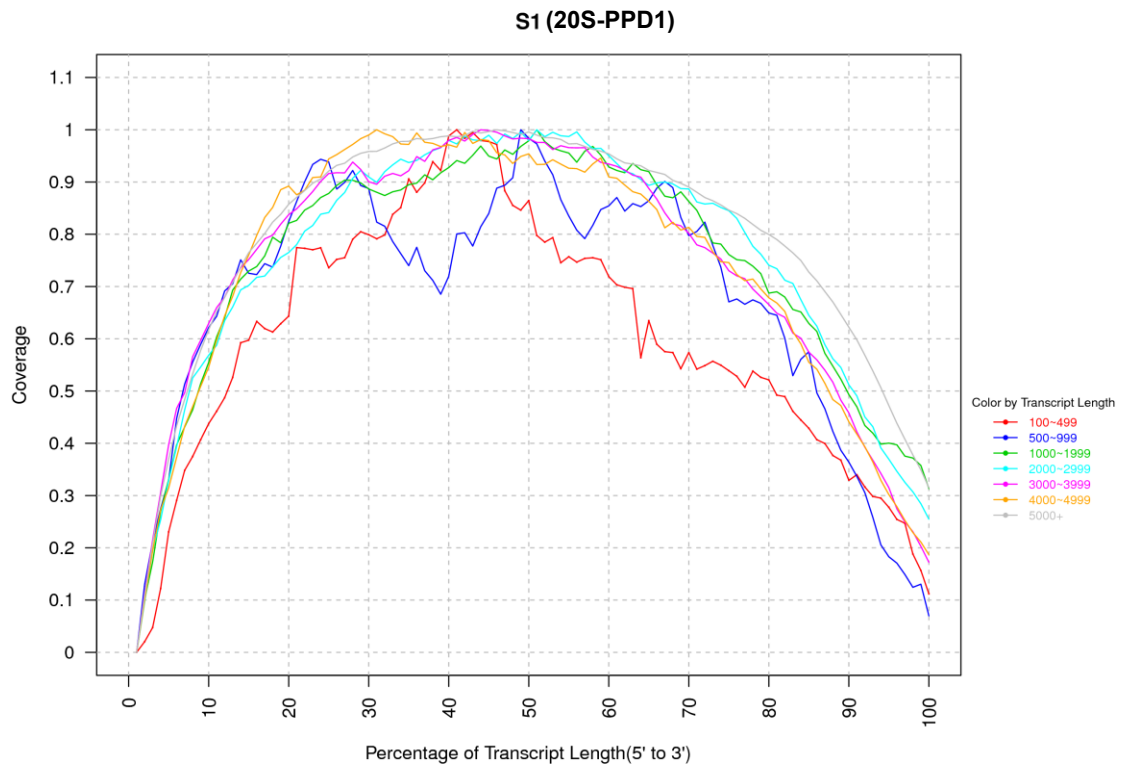

**S2 (20S-PPD2)**

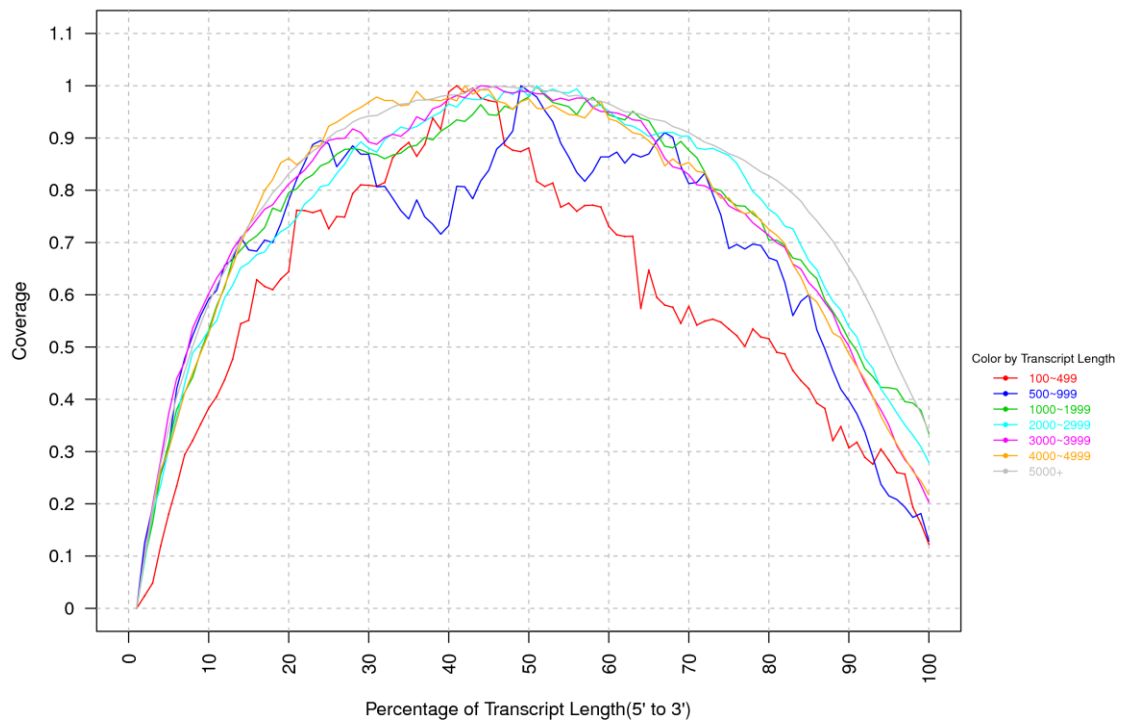

**S3 (20S-PPD3)**

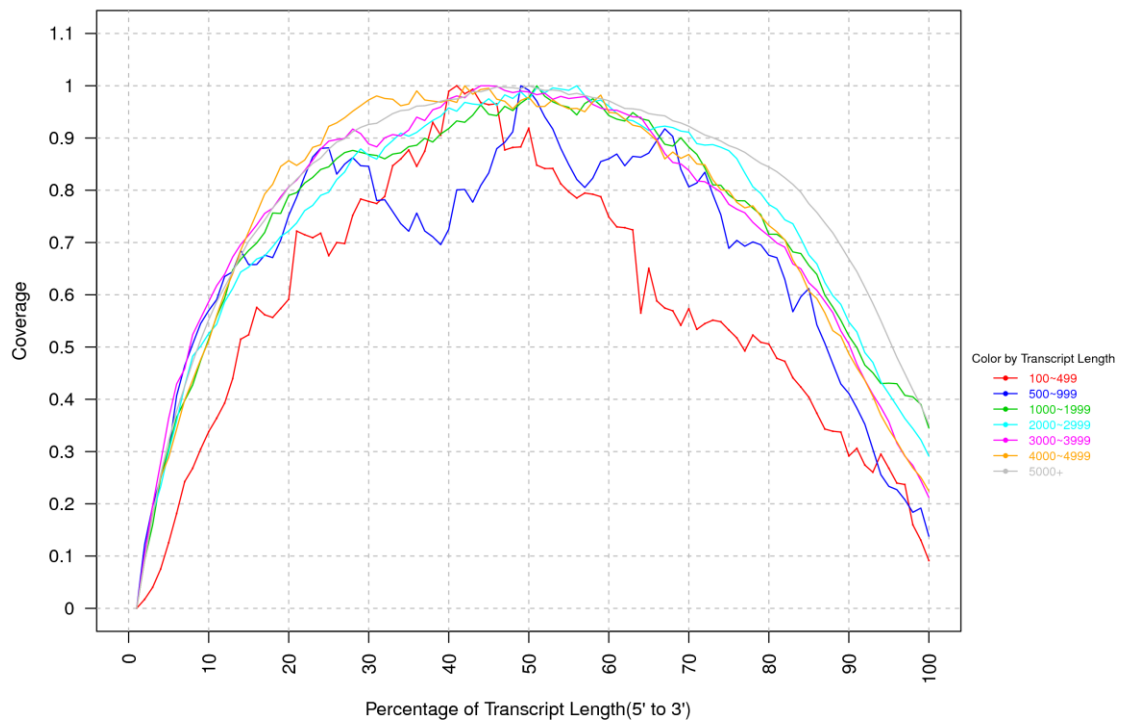

Supplement: Supplementary file 1 [file molecules-28-05746-s001.zip › Figure S5 Sequencing depth of RNA sequencing.pdf]
